# Evolution of Metabolic Phenotypes of Obesity in Coronary Patients after 5 Years of Dietary Intervention: From the CORDIOPREV Study

**DOI:** 10.3390/nu13114046

**Published:** 2021-11-12

**Authors:** Laura Martin-Piedra, Juan F. Alcala-Diaz, Francisco M. Gutierrez-Mariscal, Antonio P. Arenas de Larriva, Juan L. Romero-Cabrera, Jose D. Torres-Peña, Javier Caballero-Villarraso, Raul M. Luque, Pablo Perez-Martinez, Jose Lopez-Miranda, Javier Delgado-Lista

**Affiliations:** 1 Lipids and Atherosclerosis Unit, Internal Medicine Unit, Reina Sofia University Hospital, 14004 Córdoba, Spain; laura.martin.piedra.sspa@juntadeandalucia.es (L.M.-P.); juanf.alcala.sspa@juntadeandalucia.es (J.F.A.-D.); francisco.gutierrez@imibic.org (F.M.G.-M.); antoniop.arenas.sspa@juntadeandalucia.es (A.P.A.d.L.); juanl.romero.sspa@juntadeandalucia.es (J.L.R.-C.); h42topej@uco.es (J.D.T.-P.); pabloperez@uco.es (P.P.-M.); 2 Maimonides Institute for Biomedical Research in Cordoba (IMIBIC), 14004 Córdoba, Spain; 3 Department of Medical and Surgical Sciences, Univerisity of Cordoba, 14004 Córdoba, Spain; 4 CIBER in Physiopathology of Obesity and Nutrition, Instituto de Salud Carlos III, 28029 Madrid, Spain; bc2luhur@uco.es; 5 Biochemical Laboratory, Maimonides Institute for Biomedical Research in Cordoba, Reina Sofia University Hospital, University of Cordoba, 14004 Córdoba, Spain; bc2cavij@uco.es; 6 Maimonides Institute of Biomedical Research of Córdoba, Department of Cell Biology, Physiology and Immunology, University of Córdoba, 14004 Córdoba, Spain

**Keywords:** obesity metabolic phenotypes, metabolically healthy obese, metabolically unhealthy obese, fatty liver index, diet intervention, Mediterranean diet, low-fat diet, coronary patients

## Abstract

Background: Obesity phenotypes with different metabolic status have been described previously. We analyzed metabolic phenotypes in obese coronary patients during a 5-year follow-up, and examined the factors influencing this evolution. Methods: The CORDIOPREV study is a randomized, long-term secondary prevention study with two healthy diets: Mediterranean and low-fat. All obese patients were classified as either metabolically healthy obese (MHO) or metabolically unhealthy obese (MUO). We evaluated the changes in the metabolic phenotypes and related variables after 5 years of dietary intervention. Results: Initially, 562 out of the 1002 CORDIOPREV patients were obese. After 5 years, 476 obese patients maintained their clinical and dietary visits; 71.8% of MHO patients changed to unhealthy phenotypes (MHO-Progressors), whereas the MHO patients who maintained healthy phenotypes (MHO-Non-Progressors) lost more in terms of their body mass index (BMI) and had a lower fatty liver index (FLI-score) (*p* < 0.05). Most of the MUO (92%) patients maintained unhealthy phenotypes (MUO-Non-Responders), but 8% became metabolically healthy (MUO-Responders) after a significant decrease in their BMI and FLI-score, with improvement in all metabolic criteria. No differences were found among dietary groups. Conclusions: A greater loss of weight and liver fat is associated with a lower progression of the MHO phenotype to unhealthy phenotypes. Likewise, a marked improvement in these parameters is associated with regression from MUO to healthy phenotypes.

## 1. Introduction

Obesity is a worldwide public concern due to the effects it has on health and its socio-economic implications. Moreover, this condition continues to be on the rise worldwide [1]. Obesity is related to the most prevalent chronic diseases, such as metabolic syndrome, the risk of type 2 diabetes, cardiovascular disease (CVD), cancer or liver dysfunction, among others, and it has been proved that it is related to a reduction in life expectancy and quality of life [2]. Although obesity has been linked to all the above conditions, not all obese subjects present the same cardiometabolic risk [3,4]. Diverse hypotheses have been proposed to explain this fact, such as ectopic fat distribution [5] or adipose tissue dysfunctions [6], although the classification by body size phenotypes [7] is currently one of the most commonly cited approach.

Obesity phenotypes classify individuals by means of a combination of BMI and metabolic status. Obese individuals with a favorable metabolic profile present a phenotype which is called “metabolically healthy obese” (MHO), which has been widely studied [8], although it remains unclear whether MHO is protective against long-term obesity-related complications or is a transient phenotype. The prevalence of MHO varies widely because currently there is a lack of standardized definitions, and different prevalence reports may reflect different classifications [9]. The other phenotype of obesity is “metabolically unhealthy obese” (MUO), which is characterized by its association with risk factors and complications of obesity (diabetes, hypertension, CVD and all-cause mortality) [10]. Although this has not been clearly stated, many authors consider that MUO is the final status of all obese patients [11,12]. Some publications suggest that impairments of insulin sensitivity and low-grade subclinical inflammation, both triggered by an abnormal body fat distribution, are the most important underlying mechanisms that induce the progression from MHO to MUO [8,9,13]. In this context, body fat distribution and composition have attracted a lot of recent interest. Furthermore, amino acid metabolism and an increase in oxidative stress metabolites have been found to be altered in dysfunctional adipose tissue [14,15]. In contrast, ectopic fat, particularly liver fat, has been proposed as a key driver of cardiometabolic risk and largely explains the link between obesity and metabolic diseases, such as type 2 diabetes [5,16].

Although there has been a consistent number of studies evaluating the comparative effects of both types of obesity on clinical and biochemical variables, only a few studies have examined the long-term changes in obesity phenotypes, the factors associated with these changes, and the comparative effects of different dietary interventions [17,18,19].

Some studies have analyzed lifestyle interventions to control changes in metabolic status in the short/medium-term. Diet programs aimed at losing weight were seen to be associated with improvements in cardiometabolic risk factors in MHO individuals [20]. Moreover, changes in diet composition, such as the use of a low-fat diet (LFD) or a Mediterranean diet (MD), were efficient in reducing liver fat content and decreasing individual metabolic risk [21,22].

Morbidity rates in MUO are higher than in the MHO group. However, there is still controversy about the metabolic risk of healthy obese individuals over time [9,23]. A long-term study of MHO, evaluating their future evolution, is therefore of interest. The same applies for MUO regarding their reversion to healthy phenotypes, although their cure rate of metabolic abnormalities has been reported as low in general [22].

There are limited data in the medical literature about the characteristics of obesity phenotypes in coronary patients, their mid- and long-term evolution, and whether dietary interventions have any effect on their evolution. In the present study, we aimed to analyze changes in the two obesity phenotypes (transitions from MHO to unhealthy phenotypes and MUO to healthy phenotypes) after 5 years of follow-up in cardiovascular patients within a dietary intervention study, and to study possible differences in these phenotype transitions using two healthy dietary models (low-fat and Mediterranean diets).

## 2. Materials and Methods

### 2.1. Study Design and Study Population

The Coronary Diet Intervention with Olive Oil and Cardiovascular Prevention (CORDIOPREV) study (Clinical Trials registry NCT00924937) is a randomized, controlled dietary intervention study, involving patients undergoing secondary cardiovascular prevention. The participants (*n* = 1002) followed two different dietary patterns (MD and LFD), along with conventional treatment for coronary disease and strict follow-up protocols based on dietary and clinical monitoring. Patients were selected if they were between 20–75 years old (inclusive), had a history of established cardiovascular heart disease (but were free of events in the last six months), were willing to follow a long-term dietary intervention, and had no severe disease or a life expectancy shorter than the length of the study. A more detailed explanation of the CORDIOPREV Study has been published elsewhere [24].

The study, which follows the Helsinki declaration and the charter of good clinical practices, was approved by the Ethics Committee for Clinical Investigations of the Reina Sofia University Hospital.

### 2.2. Cardiometabolic Criteria and Metabolic Phenotypes

Metabolic health is defined as having less than 2 cardiometabolic abnormalities, as proposed by Wildman et al. [25]. Cardiometabolic abnormalities were components of metabolic syndrome (excluding waist circumference), with two additional criteria: insulin resistance and systemic inflammation (Supplementary Table S1). For the homeostasis model assessment of insulin resistance (HOMA-IR), we used adapted cut-off points for the Spanish population [26], and for the high-sensitivity C-Reactive Protein (hs-CRP), we used the cut-off point from CDC/AHA guidelines [27].

In our study, and considering the special characteristics of our population, we modified the evaluation of two Wildman criteria, not considering the intake of antihypertensive or lipid-lowering therapy as criteria, because all coronary patients should take these two treatments in the absence of contraindications, according to the treatment guidelines [28,29,30].

Given the previous definitions, in this study we analyzed two obesity phenotypes:

- Metabolically healthy obese (MHO): BMI ≥ 30 kg/m^2^ and < 2 metabolic criteria (Supplementary Materials, Table S1).
- Metabolically unhealthy obese (MUO): BMI ≥ 30 kg/m^2^ and ≥ 2 metabolic criteria (Supplementary Materials, Table S1).

### 2.3. Laboratory Tests, Anthropometric Data and Dietary Intervention

At baseline and at the yearly visits, biological samples and anthropometric measures were collected.

Fasting blood was collected into tubes with EDTA, and immediately transferred to 4 °C. Serum parameters were measured in Architect c16000 analyzers (Abbott, Chicago, IL, USA) using spectrophotometric techniques (enzymatic colorimetric methods). HOMA-IR was derived from fasting glucose and insulin levels ((fasting plasma glucose (mmol/L) × fasting serum insulin (µU/mL)/22.5). Hs-CRP was determined by means of high-sensitivity ELISA (BioCheck, Inc., Foster City, CA, USA). All samples were collected and analyzed in the Reina Sofia University Hospital in Cordoba, Spain. High levels of hs-CRP (≥10 mg/L) were excluded from the analysis to avoid nonspecific inflammation [13].

Weight and height were measured using calibrated scales (BF511 Body Composition Analyzer/Scale, OMROM, Kyoto, Japan) and a wall-mounted stadiometer (Seca 242, Health Check Systems, Brooklyn, NY, USA), respectively.

Liver fat depots were assessed using the fatty liver index (FLI), which was calculated using the following variables: triglycerides, BMI, GGT and waist circumference. Values of FLI under 30 were considered normal and those above 60 were considered as excessive liver fat [31].

The two dietary intervention models used in the CORDIOPREV studies were: (1) a Mediterranean diet composed of a minimum of 35% of total calories from fat (22% monounsaturated fatty acids-MUFAs, 6% polyunsaturated fatty acids-PUFAs, <10% saturated fatty acids-SFAs), ≤50% from carbohydrates and 15% from protein, or (2) a low-fat diet comprising <30% of total fat (12–14% MUFAs, 6–8% PUFAs, <10% SFAs), ≥55% from carbohydrates and 15% from protein. There was no total caloric restriction or weight loss intervention. Every 6 months, the patients had an individual face-to-face visit with dietitians for the assessment of dietary intake and adherence, with an additional phone contact. More information about dietary intervention has been provided elsewhere [24,32].

### 2.4. Statistical Analysis

Any variables which were skewed were transformed using decimal logarithms. Baseline characteristics are expressed as mean ± standard error of the mean (SEM) for continuous variables, and proportions (%) for categorical variables. Missing values were imputed to the closest known values in time.

According to their baseline phenotype and their 5-year metabolic health evolution, we created the following categories:

1. MHO at baseline was stratified into two groups: MHO-Non-Progressors (patients who were MHO at baseline and maintained healthy phenotypes after 5 years) and MHO-Progressors (patients who were MHO at baseline and changed to the metabolic unhealthy phenotype after follow-up).
2. MUO at baseline was divided in MUO-Responders (persons who were MUO at baseline and reversed to healthy phenotypes after follow-up) and MUO-Non-Responders (patients who were MUO at baseline and maintained the same phenotype after follow-up).

Continuous variables were compared using analysis of variance (ANOVA) and categorical variables were compared using the Chi^2^ test. To determine changes over time we used repeated-measures analysis of variance (rANOVA). Bonferroni’s test was used in the post hoc analysis. The relationship between FLI and metabolic abnormalities was tested using the Pearson correlation.

A *p*-value <0.05 was considered statistically significant. Odds ratios (OR) were represented with a confidence interval of 95% (95% CI). All statistical analyses were performed using SPSS (Statistical Package for the Social Sciences) version 24.0 for Windows (SPSS Inc., Chicago, IL, USA).

## 3. Results

### 3.1. Baseline Characteristics

A total of 1002 coronary patients were included in the CORDIOPREV study. Of these, 562 were obese at baseline, and 476 of them had complete clinical and dietary data after the 5-year follow-up (flow chart provided in Supplementary Figure S1).

Table 1 shows the baseline characteristics of the total population, stratified by MHO or MUO status. The MUO group had higher BMI, TG, HOMA-IR, hs-CRP and FLI than the MHO group at baseline. Regarding the criteria for metabolic phenotypes, hypertension (39.4% in MHO; 74.3% in MUO, *p* < 0.001) and hyperglycemia (11.3% in MHO; 76.0% in MUO, *p* < 0.001) were the most frequent abnormalities. A moderate linear correlation was observed between the initial number of abnormalities and the baseline FLI value (*r* = 0.432; *p* < 0.001). Supplementary Figure S2 shows the evolution of phenotypes of MHO and MUO after 5 years. The results of these groups are analyzed separately.

### 3.2. Evolution of MHO during Intervention with MD/LFD

At baseline, 14.9% (*n* = 71) of the subjects in this study were MHO. Mean BMI was 32.7 kg/m^2^ and they presented an ectopic fat distribution with an excess of liver fat measured via FLI (72.1 points; cut-off: 60 points) (Table 1).

#### 3.2.1. MHO Non-Progressors

In this study 28.2% of MHO subjects (*n* = 20) at baseline were classified as MHO-Non-Progressors as they remained metabolically healthy (less than two cardiometabolic abnormalities) after 5 years of follow-up. These patients decreased their BMI and improved their ectopic fat distribution, as evaluated by means of the FLI score. We found no differences in other metabolic abnormalities (Table 2).

#### 3.2.2. MHO Progressors

We classified 71.8% (51) of the MHO at baseline as MHO-Progressors, as they evolved to metabolically unhealthy phenotypes after a 5-year follow-up.

The participants in this group had decreased HDL-c concentrations and increased glucose concentrations, HOMA-IR and hs-CRP plasma levels (all *p* < 0.05). No significant changes in BMI, FLI or other metabolic abnormalities were found at the end of the study (Table 2).

#### 3.2.3. Evolution of Metabolic Abnormality Criteria in MHO Subgroups

**Figure 1:**
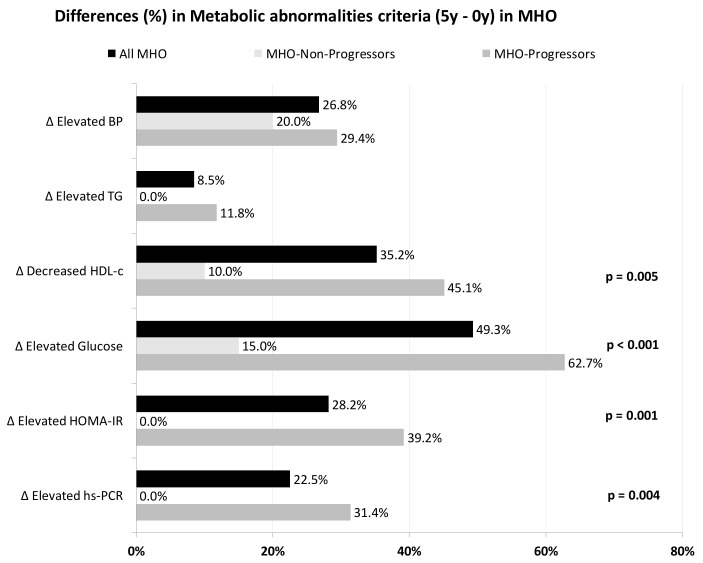
Differences in the prevalence of metabolic abnormalities (5-year minus 0-year values) in ach MHO subgroup. *p*-values are between subgroups of MHO for each abnormality. BP, blood pressure; HDL-c, high density lipoprotein-cholesterol; HOMA-IR, homeostasis model assessment of insulin resistance; hs-CRP, high sensitivity C-reactive protein; MHO, metabolically healthy obese; TG, triglycerides.

Figure 1 shows the differences in the prevalence of cardiometabolic abnormalities in MHO from baseline to the 5th year of follow-up. In the MHO-Progressors group, the percentage of patients with impaired glucose, low-HDL-c, HOMA-IR and hs-CRP increased differently to the MHO-Non-Progressors (all *p* < 0.05). More detailed data of the biochemical and anthropometric values of the different diet groups are shown in the Supplementary Materials (Table S2).

### 3.3. Evolution of MUO during Intervention with MD/LFD

At baseline, the metabolically unhealthy obese (MUO) group represented 85.1% (*n* = 405) of the obese participants. The evolution of these patients was as follows:

#### 3.3.1. MUO-Responders

After 5-years of follow-up, 8% of the subjects (*n* = 32) reversed to metabolically healthy phenotypes, classified as MUO-Responders. These patients evolved to MHO (*n* = 24; 75%), metabolically healthy overweight (MHOW; *n* = 7; 21.9%) or metabolically healthy normal weight (MHNW; *n* = 1; 3.1%) phenotypes (Supplementary Figure S2).

MUO-Responders showed an improvement in several of the variables evaluated. They had decreased BMI, triglycerides, glucose, HOMA-IR, hs-RCP and FLI scores (all *p* < 0.05) (Table 3).

#### 3.3.2. MUO-Non-Responders

We found that 92.1% of the subjects (*n* = 373) continued being metabolically unhealthy, and were labeled MUO-Non-Responders.

MUO-Non-Responders had worsened HDL-c, glucose and HOMA-IR results. Although they had decreased BMI, hs-RCP and slightly lower FLI scores, the number of cardiometabolic abnormalities per patient increased (Table 3).

#### 3.3.3. Evolution of Metabolic Abnormalities Criteria in MUO Subgroups

**Figure 2:**
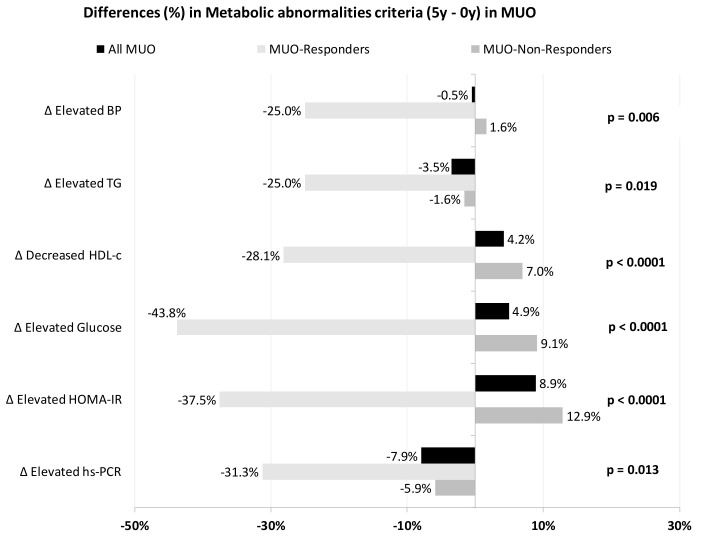
Differences in the prevalence of metabolic abnormalities (5-year minus 0-year values) in each MUO subgroup. *p*-values are between subgroups of MUO for each abnormality. BP, blood pressure; HDL-c, high density lipoprotein-cholesterol; HOMA-IR, homeostasis model assessment of insulin resistance; hs-CRP, high sensitivity C-reactive protein; MUO, metabolically unhealthy obese; TG, triglycerides.

Figure 2 represents the evolution of metabolic abnormality criteria (differences between final and baseline percentages), depending on the MUO subgroups.

MUO-Responders showed greater recovery rates in all abnormalities. More than 25% had enhanced HDL-c, insulin resistance, systemic inflammation (assessed as hs-CRP) and triglyceride levels, reaching normal values. All MUO-Responders who had baseline hypertriglyceridemia reached normalized triglyceride levels by the end of the study.

Among the MUO-Non-Responders, a significant percentage of the population had increased metabolic abnormalities, with HOMA-IR, glucose and HDL-c being the abnormalities in which we saw a greater increase in the percentage of patients suffering from them. Notably, there were no increases in the percentage of patients with altered hs-RCP or TG criteria and only a 1.6% increase for hypertension.

When we compared the evolution of both groups, significant differences were found between MUO-Responders and MUO-Non-Responders in recovery rates for blood pressure, TG, HDL-c, glucose, HOMA-IR and hs-CRP.

In MUO-Non- Responders, participants who consumed LFD lowered their BMI more than those in the MD group (LFD: 34.2 ± 0.3–33.4 ± 0.3 kg/m^2^; MD: 34.6 ± 0.3–34.5 ± 0.4 kg/m^2^; *p* = 0.011). SBP decreased more with LFD than MD (LFD: 144.0 ± 1.3–139.7 ± 1.5 mmHg; MD: 140.4 ± 1.3–143.3 ± 1.3 mmHg; *p* = 0.001). More detailed data regarding the biochemical and anthropometric values of the different diet groups are shown in the Supplementary Data (Table S3).

## 4. Discussion

Our main findings suggest that, despite the fact that most MHO patients evolved to MUO status, and that MUO is quite a stable phenotype, it is possible to slow down the natural evolution of a significant proportion of MHO to unhealthy phenotypes or even reverse, in some cases, the disease status of MUO patients over time. To our knowledge, this is the first study to examine the long-term (5-year) evolution and transitions between phenotypes of a large population of coronary patients (more than 400) in a dietary intervention without a calorie restriction.

The reported proportions of MHO and MUO in the different cohorts studied varied widely (between 7–75%) [3], due to a lack of consensus criteria for defining obesity phenotypes [8,9]. Some of the European cohorts report a proportion of MHO patients between 12% and 22%, similarly to our findings (15.8%), despite the fact that they included the general population, not patients with CVD [33], although these values are slightly lower than studies in Spanish populations, which range from 16.1% to 21.8% [22,34]. Therefore, our study suggests that the prevalence of MHO in CVD patients is no different than that of the general population and may serve as a guideline for future studies.

In our study, both MHO and MUO had similar demographic characteristics at baseline in terms of age, gender and previous episodes of CVD. As expected, there were significant differences in the diagnostic criteria of metabolic abnormalities [7,35,36], which may reflect adipose tissue dysfunction, such as adipocyte hypertrophy [9,37], immune cell alteration in adipose tissue [38], increased free fatty acids [25], oxidative stress markers or kynurenine, a precursor of diabetogenic substances [14,15], and decreased adiponectin [39].

Another suggested factor underlying the existence of MUO is an alteration in the distribution of ectopic fat, especially liver fat [9,40,41]. Although we found that the two phenotypes of obesity (MHO and MUO) had increased FLI at baseline, we noted a higher level of this score in MUO (*p* < 0.001), thus supporting this idea. We observed a linear correlation between the initial number of abnormalities and the baseline value of FLI, reinforcing the theory that the accumulation of hepatic fat is an inducer of metabolic dysfunctions (adiposopathy) [42,43].

In our study, MHO accounted for less than 15% of the obese population at baseline, and of these, about 70% lost their metabolic health status during the 5-year follow-up. This could support the idea that the MHO phenotype is mostly a transient state of which the natural evolution is its conversion into MUO [10,11,44]. However, one of the most interesting results of our study was the fact that up to 30% of the coronary patients, initially characterized as MHO, remained healthy five years later. We believe that this result is promising and may be an encouraging argument to achieve a higher engagement of these MHO patients in following a healthy diet on a long-term basis [22]. In concordance with other studies, underlying changes linked to a better prognosis were weight loss, and, perhaps more specifically, an important FLI improvement [34,45]. In contrast with other studies [46], we found no increase in the BMI of the MHO patients who progressed to MUO, but we found a lower improvement in the FLI (2% vs. 18%), accompanied by a worsening of HDL-c, glucose, HOMA-IR and PCR levels. Whether the liver fat is a cause or a consequence of the deterioration of these variables is not possible to state within the frame of this study, although the causality of liver fat accumulation as the start of an inflammatory cascade and lipotoxicity has been previously suggested in other settings [16,43,47]. In our study, the transition of MHO to unhealthy phenotypes through an increase in inflammation and insulin resistance is supported by the impairment in HOMA-IR and hs-PCR shown in patients who made that transformation. These factors could be in turn responsible for the deterioration of the glycemia found in these patients, as other authors have also suggested [6,8,9,48].

Another key question that our study aimed to evaluate was whether MUO is a permanent state, or was reversible over time. At baseline, MUO was the most common phenotype in our population (85.1%), and the metabolic disease state of most of these patients remained stationary during follow-up, indicating the relatively stable state of this phenotype, as other studies have suggested [9]. Nevertheless, and perhaps more interestingly, after clinical follow-up and close dietary intervention, 8% of these patients were able to improve some of their abnormalities and move to a metabolically healthy phenotype (MUO-Responders), even in our setting, without energy-restriction dietary recommendations. Some of the factors influencing the restoration of metabolic health in our population may be due to the baseline situation. Although we did not find differences in BMI and FLI at baseline, we did find fewer abnormalities with a lower severity in the patients who would eventually lose their metabolic disease and returned to a healthy state compared to those who remained unhealthy [9]. Some of the other differential facts when evaluating the different evolution were that MUO-Responders had a greater decrease in BMI, achieved better FLI values and showed a better HOMA-IR response to the intervention than MUO-Non-Responders. Other metabolic variables showed similar behavior, which could be explained by the reversal produced in adipose tissue dysfunction in MUO-Responders and for which dietary intervention was probably decisive [22,32].

In the MUO-Non-Responders group, the greater decrease in BMI observed in patients on the LF diet may be due to the lower final caloric intake observed in this group, as already reflected in other previous studies [32].

In summary, we have shown that, among coronary patients in a long-term dietary intervention, those with an MHO phenotype at baseline tend to transform to unhealthy phenotypes during a 5-year follow-up, and that most MUO at baseline remained unhealthy after this period. This would suggest that, in most cases, MHO converts to MUO, the latter being a stable, unhealthy state. However, up to one-third of patients with MHO can maintain this healthier phenotype for at least 5 years when they follow a strict dietary intervention with a healthy diet. Furthermore, up to one in ten patients who were MUO at baseline were able to revert to a metabolic healthy status, which reinforces the importance of dietary interventions as powerful tools to lower cardiovascular risk in these patients.

### Strengths and Limitations

One limitation of our study is the lack of a group without dietary intervention, although in patients undergoing cardiovascular secondary prevention, it would be unethical to have one. There was also an excessive representation of the male sex, derived from the natural epidemiology of coronary heart disease. Nevertheless, few studies in this disease with a five-year follow-up adhering to a strict dietary intervention have contained more than one hundred women.

As for the strengths, this study was based on a clinical dietary intervention trial with a broad sample and a long-term follow-up. Although our results cannot be extrapolated to the rest of MHO and MUO patients without CVD, to the best of our knowledge, this is the first large-scale, long-term (5-year) study of dietary intervention without calorie restriction, on the evolution of obesity phenotypes in the cardiovascular secondary prevention population.

## 5. Conclusions

In this large-scale, long-term, dietary intervention study without energy restriction in coronary patients, we found that most obese persons presented an MUO phenotype at baseline and remained in that state during the 5 years of follow-up. Likewise, most of the MHO participants at baseline transformed into unhealthy phenotypes during follow-up. However, one in ten MUO patients reversed their metabolic disease, and one-third of MHO participants at baseline even kept their healthy status. In both cases, better evolution, greater weight loss and a greater improvement in liver fat were observed, as assessed using the fatty liver index. In addition, the Mediterranean and low-fat diets performed equally well in this study.

## Figures and Tables

**Table 1:** Baseline characteristics of obese participants with 5-year follow-up. (CORDIOPREV study).

	MHO	MUO	*p*-Value
Participants (%)	71 (14.9)	405(85.1)	
Age (years)	60.3 (1.1)	59.4 (0.4)	NS
Sex (% men)	81.7	82.2	NS
Anthropometrics BMI (kg/m^2^)	32.7 (0.3)	34.4 (0.2)	<0.001
Blood pressure			
Systolic blood pressure (mmHg)	129.3 (2.0)	141.5 (1.0)	<0.001
Diastolic blood pressure (mmHg)	71.9 (1.1)	78.8 (0.6)	<0.001
Lipid profile			
Triglycerides (mg/dL)	91.8 (3.2)	149.6 (3.3)	<0.001
HDL-c (mg/dL)			
Men	46.2 (1.0)	39.0 (0.5)	<0.001
Women	57.5 (1.9)	43.9 (1.3)	<0.001
Other metabolic variables			
Glucose (mg/dL)	90.7 (1.2)	120.2 (1.8)	<0.001
Insulin (µIU/mL)	7.7 (0.5)	12.6 (0.5)	<0.001
HOMA-IR ^a^	1.7 (0.1)	3.8 (0.2)	<0.001
hs-CRP (mg/L)	1.7 (0.2)	2.9 (0.1)	<0.001
Liver function			
Fatty liver index (FLI)	72.1 (1.9)	85.0 (0.6)	<0.001
Number of metabolic abnormalities	0.8 (0.1)	3.5 (0.1)	<0.001
Metabolic abnormalities			
1. Elevated blood pressure: SBP/DBP ≥130/85 mmHg (%)	39.4	74.3	<0.001
2. Elevated triglycerides: fasting triglycerides >150 mg/dL (%)	1.4	40.5	<0.001
3. Decreased HDL-c: HDL-c <40 mg/dL (men) or <50 mg/dL (women) (%)	8.5	60.7	<0.001
4. Elevated glucose: fasting glucose ≥100 mg/dL and/or use of antidiabetics medication (%)	11.3	76.0	<0.001
5. Insulin resistance: HOMA-IR >2.6 (%)	7.0	59.8	<0.001
6. Systemic inflammation: hs-CRP ≥3 mg/L (%)	9.9	40.0	<0.001

Values are mean ± SEM, participants (%) or percentages. There are no statistically significant differences between the diet subgroups. ^a^ HOMA-IR = (Fasting plasma glucose (mmol/L) Fasting insulin (mIU/L))/22.5. BMI, body mass index; DBP, diastolic blood pressure; HDL-c, high-density lipoprotein-cholesterol; HOMA-IR, homeostasis model assessment of insulin resistance; hs-CRP, high sensitivity C-reactive protein; MHO, metabolically healthy obese; MUO, metabolically unhealthy obese; SBP, systolic blood pressure; SEM, standard error of the mean.

**Table 2:** Evolution of metabolically healthy obese participants, according to whether or not they progressed to metabolically unhealthy phenotypes.

	MHO Non-Progressors			MHO Progressors			*p*-Value
	0 Years	5 Years	*p*-Value	0 Years	5 Years	*p*-Value	Inter Groups *
Participants, *n* (%)	20 (28.2)			51 (71.8)			
Anthropometrics							
BMI (kg/m^2^)	32.9 (0.6)	31.1 (0.9)	0.033	32.6 (0.3)	32.2 (0.4)	NS	NS
Blood pressure							
Systolic blood pressure (mmHg)	131.3 (3.4)	129.8 (3.9)	NS	128.5 (2.5)	133.9 (2.7)	0.057	NS
Diastolic blood pressure (mmHg)	70.2 (2.1)	71.8 (1.6)	NS	72.5 (1.3)	74.3 (1.5)	NS	NS
Lipid profile							
Triglycerides (mg/dL)	89.9 (6.7)	80.7 (5.7)	NS	92.6 (3.6)	104.2 (6.9)	NS	NS
HDL-cholesterol (mg/dL)	51.7 (2.1) †	50.7 (2.0)	NS	47.0 (1.1) †	42.2 (1.2)	<0.001	0.048
Other metabolic variables							
Glucose (mg/dL)	89.0 (1.6)	93.3 (4.0)	NS ‡	91.4 (1.5)	104.8 (4.4)	<0.001	NS
Insulin (µIU/mL)	7.5 (1.0)	6.1 (0.4)	NS	7.8 (0.5)	10.8 (0.7)	<0.001	0.001
HOMA IR ^a^	1.7 (0.2)	1.4 (0.1)	NS	1.7 (0.1)	2.9 (0.2)	<0.001	<0.001
hs-CRP (mg/L)	1.4 (0.3)	1.0 (0.1)	NS	1.9 (0.2)	2.3 (0.2)	0.045	0.03
Liver function							
Fatty liver index (FLI)	65.8 (3.9) †	54.4 (6.3)	0.045	74.5 (2.1) †	74.1 (2.6)	NS	0.055
Number of metabolic abnormalities	0.7 (0.1)	0.8 (0.1)	NS	0.8 (0.1)	2.3 (0.1)	<0.001	<0.0001

Values are mean ± SEM or participants (%). * *p* < 0.05 in comparisons between MHO-Progressors and MHO-Non-Progressors (interaction time · MHO subgroup). † *p* < 0.05 in comparisons of variables at the beginning of the study between MHO-Progressors and MHO-Non-Progressors. ‡ *p* < 0.05 between diet subgroups. (See Supplementary Data, Table S2). ^a^ HOMA-IR = (Fasting plasma glucose (mmol/L) fasting insulin (mIU/L))/22.5. BMI, body mass index; DBP, diastolic blood pressure; HDL-c, high density lipoprotein-cholesterol; HOMA-IR, homeostasis model assessment of insulin resistance; hs-CRP, high sensitivity C-reactive protein; MHO, metabolically healthy obese; MUO, metabolically unhealthy obese; SBP, systolic blood pressure; SEM, standard error of the mean.

**Table 3:** Evolution of metabolically unhealthy obese participants, according to whether or not they reversed to metabolically healthy phenotypes.

	MUO-Responders			MUO-Non-Responders			*p*-Value Inter Groups *
	0 Years	5 Years	*p*-Value *	0 Years	5 Years	*p*-Value	
Participants, n (%)	32 (7.9)			373 (92.1)			
Anthropometrics							
BMI (kg/m^2^)	34.4 (0.7)	32.5 (0.8)	0.001	34.4 (0.2)	33.9 (0.2)	0.001 ‡	0.003
Blood pressure							
Systolic blood pressure (mmHg)	136.9 (3.3)	128.2 (2.7)	0.023	141.9 (1.1)	141.9 (0.9)	NS ‡	0.024
Diastolic blood pressure (mmHg)	77.6 (2.0)	73.7 (1.7)	NS	78.9 (0.6)	76.7 (0.6)	0.001	NS
Lipid profile							
Triglycerides (mg/dL)	118.5 (11.2) †	86.2 (4.4)	0.005	152.3 (3.4) †	146.9 (3.6)	NS	0.033
HDL-c (mg/dL)	46.0 (2.1) †	47.8 (21.5)	NS	39.4 (0.4) †	38.0 (0.5)	0.001	0.04
Other metabolic variables							
Glucose (mg/dL)	107.2 (3.8) †	93.5 (1.2)	0.001	121.3 (2.0) †	128.2 (2.2)	0.002	0.007
Insulin (µIU/mL)	9.8 (1.3)	8.0 (0.8)	NS	12.9 (0.5)	17.1 (0.8)	<0.001	0.009
HOMA IR ^a^	2.5 (0.3) †	1.9 (0.2)	0.014	3.9 (0.2) †	5.8 (0.3)	<0.001	0.012
hs-CRP (mg/L)	2.2 (0.4)	1.3 (0.2)	0.004	2.9 (0.1)	2.5 (0.1)	0.002	NS
Liver function							
Fatty liver index (FLI)	81.0 (2.9)	66.8 (4.1)	0.002	85.4 (0.6)	83.6 (0.8)	0.022	<0.0001
Number of metabolic abnormalities	2.8 (0.2) †	0.9 (0.1)	<0.001	3.6 (0.1) †	3.8 (0.1)	0.001	<0.0001

Values are mean ± SEM or participants (%). * *p* < 0.05 in comparisons between MUO-Responders and MUO-Non-Responders (interaction time MUO subgroup). † *p* < 0.05 in comparisons of variables at the beginning of the study between MUO-Responders and MUO-Non-Responders. ‡ *p* < 0.05 between diet subgroups. (See Supplementary Data, Table S3). ^a^ HOMA-IR = (Fasting plasma glucose (mmol/L) Fasting insulin (mIU/L))/22.5. BMI, body mass index; DBP, diastolic blood pressure; HDL-c, high density lipoprotein-cholesterol; HOMA-IR, homeostasis model assessment of insulin resistance; hs-CRP, high sensitivity C-reactive protein; MHO, metabolically healthy obese; MUO, metabolically unhealthy obese; SBP, systolic blood pressure; SEM, standard error of the mean.

## Data Availability

Some or all datasets generated during and/or analyzed during the current study are not publicly available but are available from the corresponding author on reasonable request.

## Supplementary Materials

The following are available online at https://www.mdpi.com/article/10.3390/nu13114046/s1, Figure S1: Flowchart of participants from the CORDIOPREV study included in the present analysis. Figure S2: Evolution of metabolic obesity phenotypes in obese cardiovascular patients after 5-year follow-up. Table S1: Metabolic abnormality criteria to define metabolic health status (Wildman). Table S2: Differences between values of analyzed variables at 5 years and at 0 years, for the MHO phenotype, according to whether or not they progressed to metabolically unhealthy phenotypes, stratified by intervention diet. Table S3: Differences between values of analyzed variables at 5 years and at 0 years, for the MUO phenotype, according to whether or not they reversed to metabolically healthy phenotypes, stratified by intervention diet.
