# Supplementary material for: Evolution of Metabolic Phenotypes of Obesity in Coronary Patients after 5 Years of Dietary Intervention: From the CORDIOPREV Study
Source: Nutrients. 2021 Nov 12;13(11):4046. doi: 10.3390/nu13114046 (PMC8624211; doi:10.3390/nu13114046)
Supplement: Supplementary file 1 [file nutrients-13-04046-s001.zip › nutrients-1413515-supplementary.pdf]

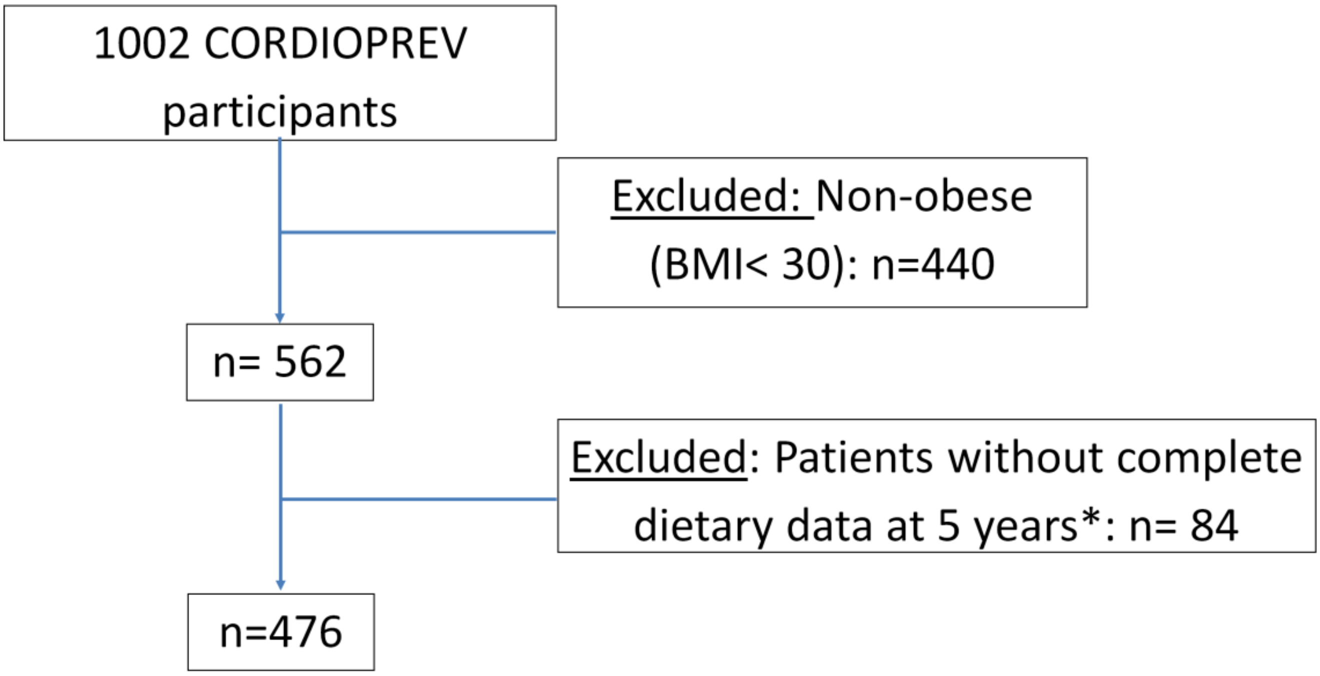

Supplementary Figure S1: Flowchart of participants from the CORDIOPREV study included in the present analysis.
*This value includes those patients who died during the 5-year study, and those who had discontinued the dietary intervention at 5 years.

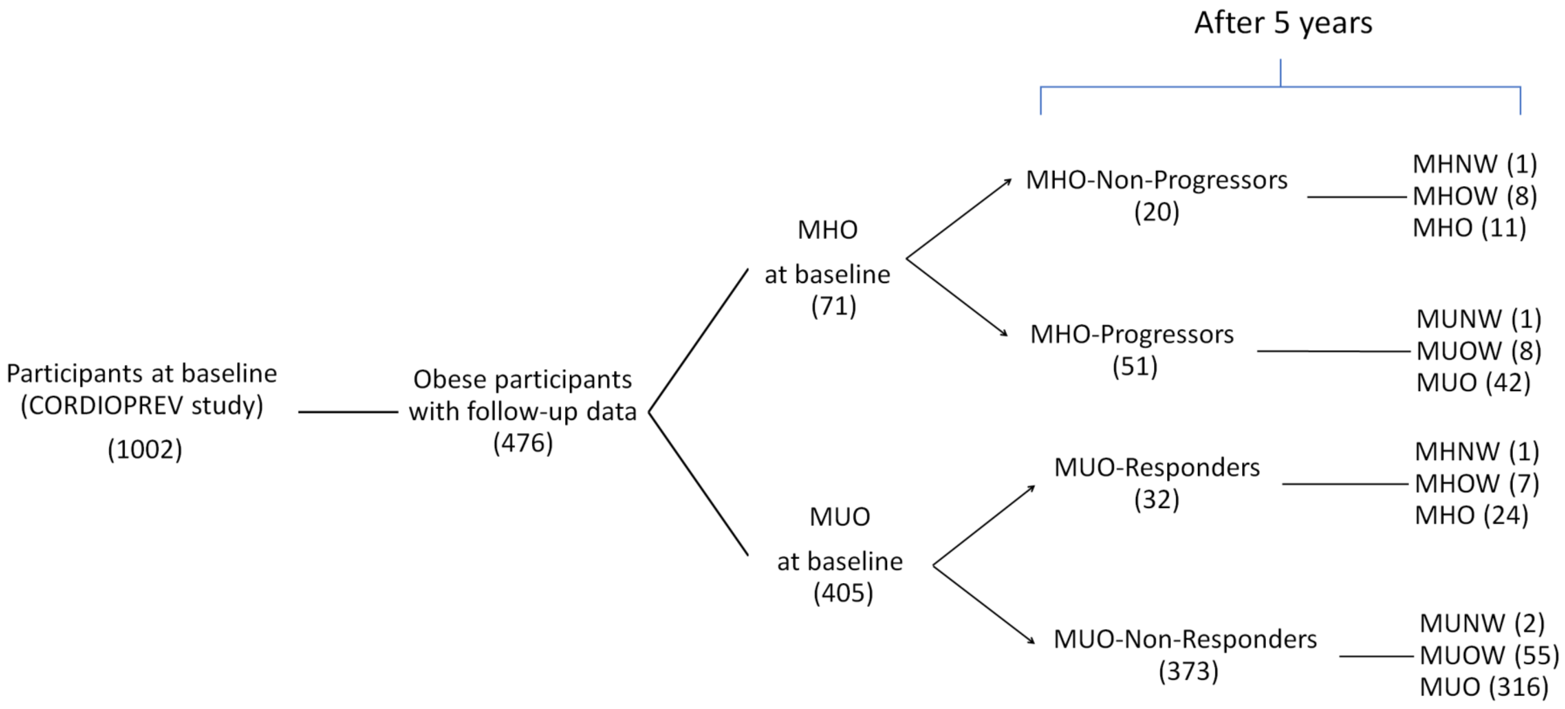

Supplementary Figure S2: Evolution of metabolic obesity phenotypes in obese cardiovascular patients after 5-year follow-up.
Obesity phenotypes: MHNW, Metabolically Healthy Normal Weight; MHOW, Metabolically Healthy Overweight; MHO, Metabolically Healthy Obese; MUNW, Metabolically Unhealthy Normal Weight; MUOW, Metabolically Unhealthy Overweight; MUO, Metabolically Unhealthy Obese.

Supplementary Table S1: Metabolic abnormalities criteria to define metabolic health status (Wildman)(23)

1 Elevated blood pressure: systolic/diastolic blood pressure ≥130/85 mmHg or antihypertensive medication use
2 Elevated triglyceride level: fasting triglyceride level ≥150 mg/dL
3 Decreased HDL-c level: HDL-c level <40 mg/dL in men or <50 mg/dL in women or lipid-lowering medication use
4 Elevated glucose level: fasting glucose level ≥100 mg/dL or antidiabetic medication use
5 Insulin resistance: HOMA-IR >2.6
6 Systemic inflammation: hs-CRP level ≥3 mg/L

HDL-c, High Density Lipoprotein-cholesterol; HOMA-IR, Homeostasis Model Assessment of Insulin Resistance; hs-CRP, high sensitivity C-Reactive Protein

Supplementary Table S2: Differences between values of analyzed variables at 5 years and at 0 years, for the MHO phenotype, according to whether or not they progress to metabolically unhealthy phenotypes, stratified by intervention diet.

	MHO-Non-Progressors (n=20)			MHO-Progressors (n= 51)		
	LFD	MD	p-value* inter groups	LFD	MD	p-value* inter groups
**Participants, n (%)**	**8 (40.0)**	**12 (60.0)**	NS	**20 (39.2)**	**31 (60.8)**	NS
Δ BMI (kg/m^2)	-2.4 (1.7)	-1.3 (0.6)	NS	-0.1 (0.6)	-0.7 (0.5)	NS
Δ SBP (mmHg)	4.0 (9.6)	-5.3 (3.6)	NS	3.7 (4.4)	6.5 (3.5)	NS
Δ DBP (mmHg)	3.8 (3.8)	0.2 (2.6)	NS	2.1 (2.3)	1.5 (2.3)	NS
Δ TG (mg/dL)	-8.6 (11.6)	-9.6 (8.2)	NS	19.1 (8.2)	6.7 (11.1)	NS
Δ HDL-c (mg/dL)	0.6 (2.7)	-2.0 (1.7)	NS	-7.0 (1.3)†	-3.4 (1.5)†	NS
Δ Glucose (mg/dL)	15.4 (7.9)†	-3.2 (2.8)	**0.019**	16.5 (6.9)†	11.5 (3.3)†	NS
Δ Insulin (μIU/mL)	-1.2 (0.9)	-1.6 (1.5)	NS	2.2 (1.0)†	3.5 (0.9)†	NS
Δ HOMA IR [a]	-0.1 (0.2)	-0.4 (0.4)	NS	1.0 (0.4)†	1.2 (0.3)†	NS
Δ hs-CRP (mg/L)	-0.8 (0.7)	-0.2 (0.2)	NS	0.5 (0.4)	0.4 (0.2)	NS
Δ FLI	-9.3 (7.5)	-11.0 (6.4)	NS	3.3 (2.9)	-3.5 (3.7)	NS
Δ Metabolic abnormalities	0.3 (0.3)	0.2 (0.1)	NS	2.1 (0.2)†	2.0 (0.2)†	NS

Values indicate differences (Δ) in the analyzed variables, value (5 years) minus value (0 years), for each diet group, expressed as mean ± SEM, or number of participants (%).

* $p<0.05$ in comparisons between diet subgroups, using repeated measures ANOVA (time · diet interaction).

† $p<0.05$ between values of each variable at 0 years and 5 years, using Repeated Measures ANOVA, for each diet subgroup.

[a] HOMA-IR= [Fasting plasma glucose (mmol/L) · Fasting insulin (mIU/L)] /22.5

BMI, Body Mass Index; DBP, Diastolic Blood Pressure; FLI, Fatty Liver Index; HDL-c, High Density Lipoprotein-cholesterol; HOMA-IR, Homeostasis Model Assessment of Insulin Resistance; hs-CRP, high sensitivity C-Reactive Protein; LFD, Low-Fat Diet; MD, Mediterranean Diet; MHO, Metabolically Healthy Obese; SBP, Systolic Blood Pressure; SEM, Standard Error of the Mean.

Supplementary Table S3: Differences between values of analyzed variables at 5 years and at 0 years, for the MUO phenotype, according to whether or not they reverse to metabolically healthy phenotypes, stratified by intervention diet.

	**MUO-Responders (n=32)**			**MUO-Non-Responders (n= 373)**		
	LFD	MD	p-value* inter groups	LFD	MD	p-value* inter groups
**Participants, n (%)**	**19 (59.4)**	**13 (40.6)**		**188 (50.4)**	**185 (49.6)**	
Δ BMI (kg/m^2)	-2.0 (0.7)†	-1.8 (0.8)†	NS	-0.8 (0.2)†	-0.1 (0.2)	**0.011**
Δ SBP (mmHg)	-6.9 (5.0)	-11.3 (5.4)	NS	-3.5 (1.4)†	3.6 (1.6)†	**0.001**
Δ DBP (mmHg)	-5.7 (2.3)	-1.1 (4.2)	NS	-3.2 (0.8)†	-1.0 (0.9)	NS
Δ TG (mg/dL)	-40.4 (16.5)†	-20.4 (9.9)	NS	-2.1 (5.7)	-6.6 (4.9)	NS
Δ HDL-c (mg/dL)	1.9 (2.7)	1.6 (2.4)	NS	-1.9 (5.6)†	-0.8 (0.6)	NS
Δ Glucose (mg/dL)	-12.6 (3.0)†	-15.2 (8.4)†	NS	10.0 (3.3)†	3.1 (2.8)	NS
Δ Insulin (μIU/mL)	-2.5 (1.1)	-0.5 (1.9)	NS	3.5 (0.7)†	4.9 (1.1)†	NS
Δ HOMA IR [a]	-0.9 (0.3)†	-0.4 (0.5)	NS	1.6 (0.3)†	1.7 (0.4)†	NS
Δ hs-CRP (mg/L)	-0.7 (0.2)	-1.2 (0.7)†	NS	-0.3 (0.2)†	-0.4 (0.2)†	NS
Δ FLI	-16.3 (5.1)†	-12.4 (7.4)	NS	-2.7 (1.0)†	-0.4 (0.9)	NS
Δ Metabolic abnormalities	-1.9 (0.2)†	-1.9 (0.4)†	NS	0.2 (0.1)†	0.3 (0.1)†	NS

Values indicate differences (Δ) in the analyzed variables, value (5 years) minus value (0 years), for each diet group, expressed as mean (SEM), or number of participants (percentages).

* $p<0.05$ in comparisons between diet subgroups, using repeated measures ANOVA (time · diet interaction).

† $p<0.05$ between values of each variable at 0 years and 5 years, using Repeated Measures ANOVA, for each diet subgroup.

[a] HOMA-IR= [Fasting plasma glucose (mmol/L) · Fasting insulin (mIU/L)] /22.5

BMI, Body Mass Index; DBP, Diastolic Blood Pressure; FLI, Fatty Liver Index; HDL-c, High Density Lipoprotein-cholesterol; HOMA-IR, Homeostasis Model Assessment of Insulin Resistance; hs-CRP, high sensitivity C-Reactive Protein; LFD, Low-Fat Diet; MD, Mediterranean Diet; MUO, Metabolically Unhealthy Obese; SBP, Systolic Blood Pressure; SEM, Standard Error of the Mean.
